# Microglia as candidate links between hormonal signals and neuronal circuits in polycystic ovary syndrome: evidence gaps and a research agenda

**DOI:** 10.3389/fendo.2026.1952469

**Published:** 2026-10-02

**Authors:** Hongtao Li, Tong Yin, Yichen Wang, Yixuan Wang, Xiaoyuan Lin, Xinyan Jia, Wei Yi, Han Yang

**Affiliations:** 1 South China Research Center for Acupuncture and Moxibustion, School of Acupuncture-Moxibustion and Rehabilitation, Guangzhou University of Chinese Medicine, Guangzhou, Guangdong, China; 2 The Second Clinical Medical College, Guangzhou University of Chinese Medicine, Guangzhou, Guangdong, China; 3 Tianjin University of Traditional Chinese Medicine, Tianjin, China; 4 Shenzhen Bao'an Authentic TCM Therapy Hospital, Shenzhen, Guangdong, China; 5 School of Nursing, Guangzhou University of Chinese Medicine, Guangzhou, Guangdong, China

**Keywords:** androgen excess, evidence synthesis, gonadotropin-releasing hormone, hypothalamus, microglia, neuroendocrinology, neuroinflammation, polycystic ovary syndrome

## Abstract

Polycystic ovary syndrome (PCOS) combines reproductive endocrine dysfunction with metabolic, autonomic, and neurobehavioral phenotypes, yet the contribution of brain microglia remains uncertain. We conducted a structured narrative review that separates human PCOS evidence, PCOS-like animal findings, PCOS-related cellular observations, and non-PCOS mechanistic bridges. Only three heterogeneous PCOS-like studies directly assessed microglial outcomes, and none established a conserved microglial state, microglial necessity, a complete hormone–microglia–neuron causal chain, or human relevance. We therefore propose the PCOS Neuroimmune-Endocrine Triangular Network (PNETN), a falsifiable framework linking endocrine and metabolic signals, context-dependent microglial states, and neuronal circuits regulating GnRH/KNDy activity, luteinizing-hormone dynamics, ovulation, metabolism, autonomic function, and behavior. The framework defines criteria for causal promotion, including cell identity, regional specificity, temporal precedence, selective perturbation, rescue, orthogonal functional readouts, and independent replication. Microglia should currently be regarded as candidate contributors, not established clinical targets.

## 1 Introduction

### 1.1 PCOS as a heterogeneous, multi-system disorder

Polycystic ovary syndrome (PCOS) affects approximately 10–13% of reproductive-aged women and is characterized primarily by hyperandrogenism, ovulatory dysfunction, and polycystic ovarian morphology, with insulin resistance, adiposity, sleep disturbances, and psychological manifestations varying across phenotypes (1). This heterogeneity argues against a single mechanism operating uniformly across all patients.

PCOS is increasingly understood as a multifactorial disorder involving interactions among ovarian steroidogenesis, metabolic signaling, hypothalamic–pituitary–ovarian regulation, and genetic and developmental susceptibility. Hyperandrogenism, insulin resistance, and impaired steroid feedback can interact to alter gonadotropin secretion and ovarian function, providing a physiological link between peripheral endocrine–metabolic abnormalities and central reproductive regulation. In 2026, a multistep global consensus process established polyendocrine metabolic ovarian syndrome (PMOS) as the new name for PCOS, emphasizing its multisystem endocrine, metabolic, and ovarian pathophysiology (2). Because the present literature was largely generated under the term PCOS, we retain PCOS throughout this review for continuity with the cited evidence while acknowledging the transition to PMOS terminology.

Within this framework, the central nervous system is particularly relevant because GnRH neurons and upstream KNDy, GABAergic, and other neuronal populations integrate steroid, metabolic, and neural signals to shape gonadotropin secretion. Altered steroid feedback, neuronal input, or neuroendocrine circuit activity may therefore contribute to reproductive dysfunction in selected PCOS phenotypes. Existing reviews have described neuronal, reproductive, metabolic, and glial perspectives (3–5), but the links among hormonal signals, brain immune cells, and reproductive neuronal circuits remain incompletely defined.

### 1.2 Human studies report central phenotypes, not a microglial mediator

Human evidence is strongest for neuroendocrine and central physiological phenotypes. Multi-day steroid feedback supports reduced sensitivity of the GnRH pulse generator under a defined protocol, whereas acute progesterone exposure did not show a clear PCOS-specific defect (6, 7). Neurokinin 3 receptor antagonism altered gonadotropin output while preserving kisspeptin responsiveness (8). Small studies also report CSF GABA and estradiol differences, resting-state network changes, cerebral-autonomic coupling, and AMH-associated hypothalamic measures (9–12). These observations are clinically relevant but cell-nonspecific. In the AMH study, the mechanistic neuroglial interface demonstrated in mice involved tanycytes and GnRH terminals, not microglia (12).

The apparent discrepancies among human studies are informative rather than mutually exclusive. Multi-day steroid feedback, acute crossover exposure, sleep-stage analyses, insulin-clamp physiology, and neuropeptide challenge interrogate different time scales and physiological states (6–8, 17–21). A higher or more persistent LH signal cannot be assumed to arise from the same circuit in lean hyperandrogenic PCOS, obesity-associated PCOS, treated participants, or irregular-cycle phenotypes. These studies therefore define outputs that a cellular mechanism must explain; they do not locate the initiating cell or demonstrate a single GnRH abnormality across the syndrome.

### 1.3 Direct PCOS-microglia evidence is limited to heterogeneous preclinical studies

Direct PCOS-microglia evidence is restricted to three non-equivalent preclinical studies. Prenatal-androgenized mice showed developmental and regional microglial changes with reduced uptake of GABAergic material (13). A letrozole-rat study reported candidate GnRHR expression in hypothalamic microglia and exercise-associated endocrine and marker changes, but lacked receptor loss-of-function and did not establish microglial mediation (14). A DHT-rat/BV2 study associated troxerutin with IL-22/IL-22R1/IRF3-related readouts and GnRH-related improvement; publicly accessible information does not establish microglia-specific *in vivo* knockdown (15). These findings nominate testable nodes but do not define a shared state, prove microglial necessity, or support human efficacy.

The methodological ceilings of these three studies differ. The prenatal-androgen study was spatial and developmental but observational; the letrozole study combined tissue and cultured-cell assays without receptor-specific loss-of-function; and the DHT study evaluated a systemic intervention whose *in vivo* cell specificity is not resolved in the public abstract (13–15). Their convergence is therefore limited to the proposition that microglial measures can change in selected PCOS-like contexts. They cannot be quantitatively pooled, and a treatment-responsive marker cannot be assumed to be the disease-initiating mechanism.

### 1.4 Scope and contribution: the PNETN framework

We therefore propose the PCOS Neuroimmune-Endocrine Triangular Network (PNETN) as an evidence-graded and falsifiable organizing framework, not a demonstrated feed-forward loop. It links endocrine and metabolic exposures, context-dependent microglial states, and neuronal circuits controlling reproductive, metabolic, autonomic, and behavioral outputs. Its value lies in making evidence asymmetry visible and specifying when an association can be promoted to a mechanism: cell identity, regional localization, temporal precedence, selective perturbation, a defined effector, an orthogonal neural or endocrine endpoint, rescue, and independent replication. This contribution extends prior PCOS brain and reproductive-glia syntheses (3–5, 16, 168–171) by emphasizing inferential boundaries rather than presuming a unified microglial pathway. The overall evidence architecture and proposed PNETN framework are summarized in **Figure 1**.

**Figure 1:**
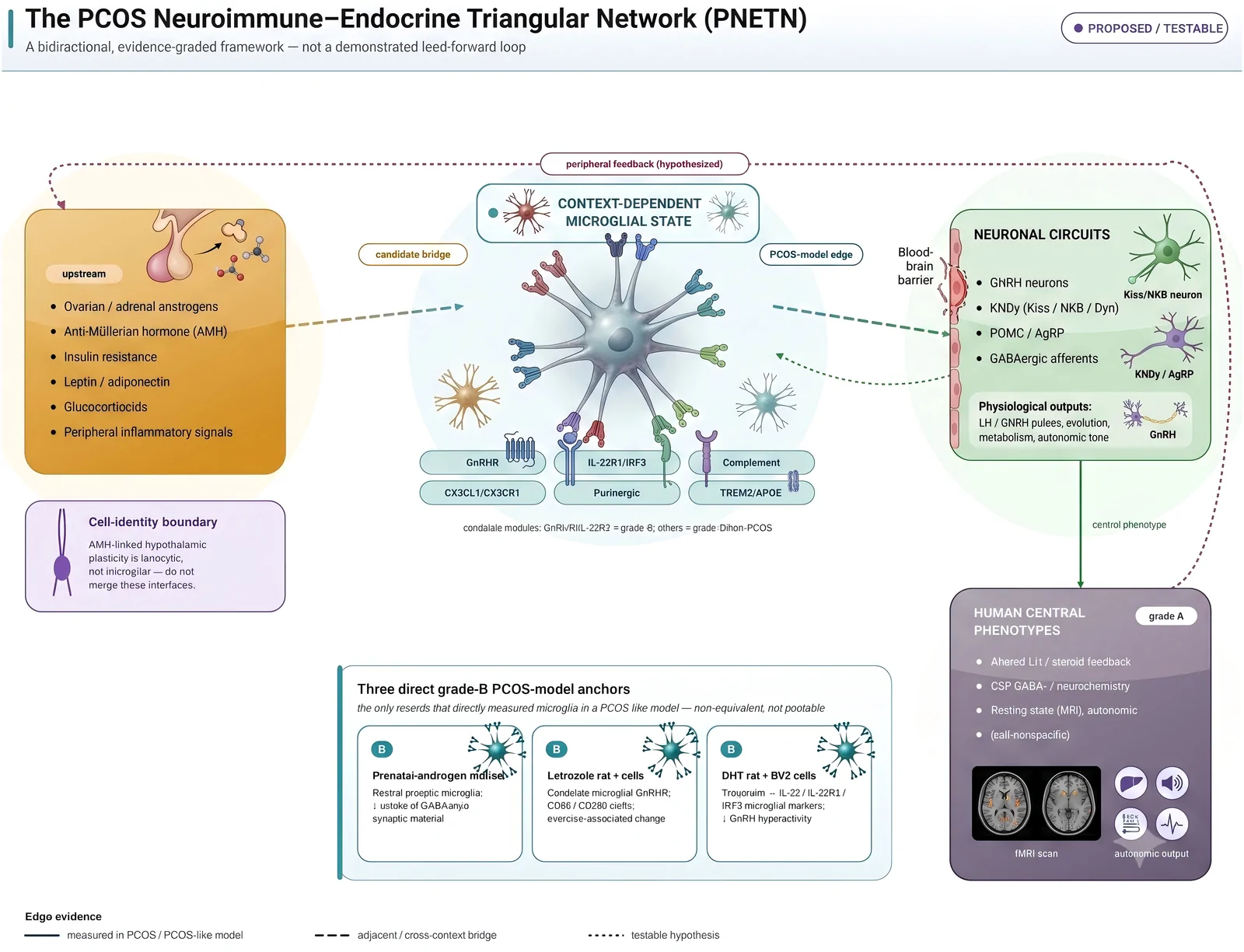
The PCOS Neuroimmune-Endocrine Triangular Network (PNETN). The diagram separates endocrine and metabolic inputs, candidate microglial sensor/effector modules, neuronal circuits, human cell-nonspecific central phenotypes, and physiological outputs. GnRHR and IL-22R1/IRF3 are labeled as grade-B PCOS-model candidates; complement, CX3CL1/CX3CR1, purinergic and TREM2/APOE modules are grade-D non-PCOS bridges. The three direct PCOS-like anchors remain separate (13–15). AMH-linked plasticity is assigned to tanycytes rather than microglia (12). Solid, dashed and dotted edges denote measured relationships, bridges and hypotheses. PNETN is proposed and testable, not an established feed-forward loop.

## 2 Review scope and evidence framework

### 2.1 Search, eligibility and reproducibility boundary

To improve transparency without retrospectively reconstructing an unavailable screening history, **Supplementary Figure 1** summarizes the search and evidence-selection framework, whereas **Supplementary Table 1** lists the studies included in the final narrative synthesis.

A structured PubMed/MEDLINE-centered narrative search was used to identify human PCOS studies, disease-relevant PCOS-like models, PCOS-related cell or tissue evidence, and clearly labeled non-PCOS mechanistic bridges through 20 July 2026. Reviews, guidelines, and consensus statements were used for framing; pivotal mechanistic claims were preferentially anchored to primary studies. Peripheral biomarkers were not accepted as evidence of brain microglial activation, ovarian immune-cell studies required a central bridge, and tanycytes, astrocytes, infiltrating myeloid cells, BV2 cells, primary microglia, and whole-hypothalamus measurements were kept distinct. Historical executable searches, complete exports, deduplication files, and dual-review screening records were not retained. The review is therefore not a systematic review, scoping review, mapping review, or PRISMA synthesis, and no reproducible screening denominator is reported.

### 2.2 Evidence grading and claim adjudication

Evidence-source grades describe proximity to the PCOS question rather than methodological quality: grade A, human PCOS; grade B, PCOS-like animal models; grade C, PCOS-related cell, tissue, or omics data; grade D, non-PCOS mechanistic bridges; and grade E, synthesis or explicit hypotheses. Directness was judged separately as Direct, Adjacent-PCOS, or Indirect. Strong causal wording was reserved for designs with an appropriate intervention or perturbation, while cross-sectional, marker, imaging, bulk-tissue, animal, and cell-line findings were bounded to their source context. Internal identifier checks and a claim-citation tracker support traceability but do not constitute a complete external authenticity, full-text, correction, expression-of-concern, or retraction audit. Bibliographic identifiers were checked where available, and final peer-reviewed versions were preferentially cited. The evidence-grading framework is summarized in **Table 1**, and sentinel records anchoring the evidence architecture are presented in **Table 2**.

**Table 1:** Evidence grade, directness and permitted wording.

Evidence grade	Operational definition	Permitted wording
A	Direct human PCOS evidence with an interpretable design	“was observed in women with PCOS”; causal verbs only when the design supports them
B	PCOS-like animal-model evidence meeting the stated disease-relevance criteria	“in this PCOS-like model”; no automatic human translation
C	Direct PCOS-related cell, tissue or omics evidence	“in the sampled tissue/cell context”; no whole-organism generalization
D	Obesity, insulin resistance, neuroinflammation, reproductive disease or general mechanistic evidence	“may provide a mechanistic bridge”; “has not been demonstrated in PCOS”
E	Synthesis, untested network edge or explicit hypothesis	“could,” “might,” “remains to be established”
Directness	Interpretation	Permitted wording
Direct	The study directly addresses its recorded PCOS or mechanistic context	“directly measured” or “directly observed” within the stated study context
Adjacent-PCOS	Relevant to PCOS but does not measure the complete target edge	“PCOS-relevant” or “adjacent evidence”; does not test the complete target edge
Indirect	Cross-disease or general biology used only as a bridge	“provides cross-context mechanistic support”; “has not been demonstrated in PCOS”

This rubric reflects evidence proximity rather than methodological quality and is not a formal risk-of-bias assessment.

**Table 2:** Sentinel records anchoring the evidence architecture.

Reference no.	Study context	Main supported claim	Evidence grade/directness	Principal limitation
(1)	International guideline	PCOS is heterogeneous across reproductive, metabolic, sleep and psychological domains	Synthesis/Adjacent-PCOS	Not mechanistic brain evidence
(6)	Human steroid-feedback physiology	Lower progesterone exposure suppressed LH pulse frequency less effectively in PCOS under a multi-day protocol	A/Direct	LH is a GnRH proxy; protocol and metabolic confounding
(7)	Human acute crossover physiology	Acute progesterone feedback did not show a clear PCOS-specific defect	A/Direct	A short exposure may not reproduce multi-day feedback
(8)	Human NKB/kisspeptin intervention	NK3R antagonism altered gonadotropin secretion and LH pulse frequency	A/Direct	Small, short study without direct KNDy recording
(9)	Human CSF study	CSF GABA and estradiol were higher in a small non-obese PCOS cohort	A/Direct	Cross-sectional, non-regional and not cell-specific
(10)	Human resting-state fMRI	Regional and network-level cerebral differences were associated with endocrine/metabolic measures	A/Direct	BOLD is indirect and correlations require replication
(11)	Human autonomic/fMRI study	Sympathoexcitation accompanied orbitofrontal BOLD differences	A/Direct	Insulin-sensitivity adjustment attenuated group differences
(12)	Human imaging plus mouse mechanism	AMH-associated human hypothalamic measures and mouse tanycyte-GnRH plasticity	A/B mixed	Human association and mouse causality must remain separate
(13)	Prenatal-androgen mouse	Developmental microglial and GABAergic-engulfment changes	B/Direct	Morphology/engulfment without causal microglial perturbation
(14)	Letrozole rat and cell assays	Candidate microglial GnRHR signaling and exercise-associated marker changes	B/Direct	No receptor loss-of-function; M1/M2 marker limitations
(15)	DHT rat and BV2 intervention	Candidate IL-22R1/IRF3-linked microglial and GnRH effects	B/Direct	Preclinical; cell specificity requires full-method confirmation
(168–171)	Prior PCOS brain, hypothalamic-inflammation and reproductive-glia reviews	Earlier syntheses define central phenotypes and plausible glial interfaces but do not provide direct human PCOS brain-microglia evidence	E/Adjacent-PCOS	Secondary synthesis; cannot substitute for cell-specific primary evidence

The first column lists in-text reference numbers, not PubMed identifiers. DOI strings are reported in the reference list; PMID identifiers are maintained in the internal bibliographic inventory.

## 3 Human neuroendocrine and central phenotypes

### 3.1 Altered LH/GnRH pulse dynamics and steroid feedback

Human studies support altered reproductive-axis regulation but not one invariant LH/GnRH pulse phenotype. Higher LH pulse amplitude, faster baseline pulses in selected lean cohorts, reduced feedback sensitivity during multi-day steroid exposure, preserved acute progesterone feedback, opioid-modulated slowing, and altered sleep-stage patterning were observed under different protocols (6, 7, 17–20). These differences make exposure duration, achieved hormone concentrations, sleep, cycle phenotype, and insulin sensitivity effect modifiers rather than nuisance variables. Circulating LH remains an indirect readout of GnRH pulse-generator activity, and none of these studies identifies a microglial mediator.

Protocol-specific null findings should remain visible. The acute progesterone crossover did not reproduce the reduced feedback sensitivity observed during longer exposure (6, 7), and insulin-associated pulse differences were reported in selected lean participants rather than an unstratified PCOS population (18). Naloxone reversal of progestin-associated slowing suggests inducible opioid modulation, whereas sleep-linked pulse initiation implicates circadian and state-dependent inputs (19, 20). These studies support a dynamic pulse generator influenced by several pathways, not a fixed lesion or a microglial diagnosis.

### 3.2 Kisspeptin-neurokinin B-GABA control of reproductive output

Human kisspeptin/NKB studies narrow the candidate circuitry. Kisspeptin-LH coupling varied by menstrual phenotype, and short-term NK3R antagonism reduced gonadotropin secretion and LH pulse frequency while preserving the kisspeptin response (8, 21). These interventions show that the NKB-kisspeptin axis can modulate reproductive output in PCOS; they do not directly record KNDy neurons or establish disease reversal.

Preclinical evidence is broader but inconsistent across levels of measurement. Prenatal androgen or testosterone exposure altered GABAergic transmission, afferent apposition, and inputs to GnRH/KNDy neurons, while targeted neuronal manipulations produced positive, null, or branch-specific reproductive effects (22–30). Synapse counts, postsynaptic currents, receptor transcripts, and circulating LH are not interchangeable. These studies define candidate neuronal circuits but do not establish a microglia-to-KNDy edge.

GABA and KNDy findings illustrate the distinction between anatomy and physiology. Increased apposition or inferred synapse number does not guarantee stronger transmission, and unchanged intrinsic firing does not exclude altered peptide release or afferent timing (24, 27, 36, 40). Arcuate GABA activation can raise LH and generate reproductive abnormalities in healthy mice, yet the acute response can differ after prenatal androgen exposure (26). Androgen-receptor deletion in selected neuronal populations produces partial or null protection (28–30, 57). These results make direct neuronal pathways important competing explanations whenever a microglial upstream role is proposed.

KNDy outcomes likewise depend on model and assay. Letrozole, prenatal androgen/DHT, antiprogestin, and DHT paradigms produced varied changes in Kiss1, Tac2, Pdyn, steroid receptors, connectivity, peptide abundance, or network output (31–36). Kisspeptin- or neuropeptide-directed interventions improved selected endpoints (37–39), whereas intrinsic KNDy firing or postsynaptic responsiveness was preserved in other designs (40, 41). Expression, connectivity, firing, peptide release, and synchrony should therefore remain separate claims.

### 3.3 Metabolic and gonadal signals at the neuroglial-endocrine interface

Metabolic and gonadal signals shape a human central receiving environment without identifying the responsible cell. CSF neurochemistry, allopregnanolone sensitivity, corticolimbic insulin responses, autonomic-associated activation, and AMH-associated hypothalamic measures vary with PCOS phenotype and metabolic context (9, 11, 12, 42–44). The AMH evidence is particularly instructive: human findings are associative, whereas the mouse structural mechanism involves tanycytes and GnRH-terminal access. It cannot be rewritten as an AMH-microglia pathway.

The same caution applies to central metabolic measures. Insulin-sensitive corticolimbic responses and cerebral-autonomic associations can be attenuated after adjustment for insulin sensitivity (11, 43). Allopregnanolone-related GABA-A sensitivity may track weight status more strongly than PCOS diagnosis (42). A mechanism that ignores adiposity, insulin resistance, cycle stage, and medication exposure risks attributing a heterogeneous metabolic-central phenotype to reproductive hormones or microglia without adequate separation.

PCOS-like models add bulk hypothalamic leptin, inflammatory, GABA, NLRP3, and methylation findings (45–49), but only three studies directly anchor a microglial node (13–15). The remaining measurements are cell-unspecified or intervention-associated and do not establish the complete endocrine-to-cell-to-neuron sequence. Human central phenotypes and the corresponding cell-identity boundary are summarized in **Figure 2**. The three direct PCOS-like microglial anchors are compared in **Figure 3**.

**Figure 2:**
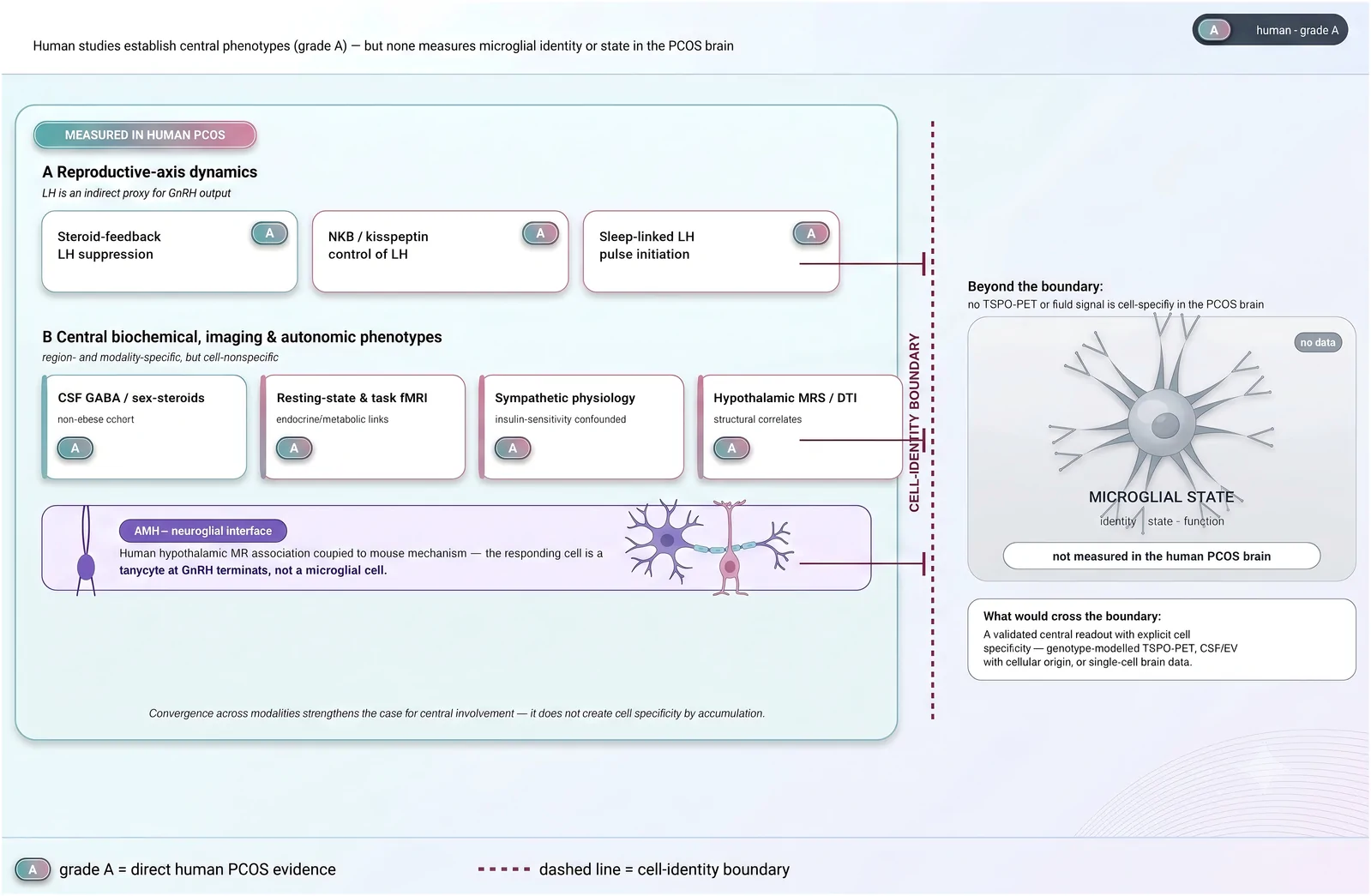
Human PCOS central phenotype map and the cell-identity boundary. Human PCOS studies report reproductive-axis physiology, central biochemical findings, imaging correlates, autonomic phenotypes, sleep-linked LH timing, and hypothalamic MRS/DTI findings (6–12, 20, 150). These observations are region- and modality-specific but cell-nonspecific. The AMH-associated neuroglial interface is tanycytic, not microglial (12, 44). The bold boundary indicates that a human central phenotype does not establish a human brain-microglial state. Grade A denotes direct human PCOS evidence, not cell specificity or methodological quality.

**Figure 3:**
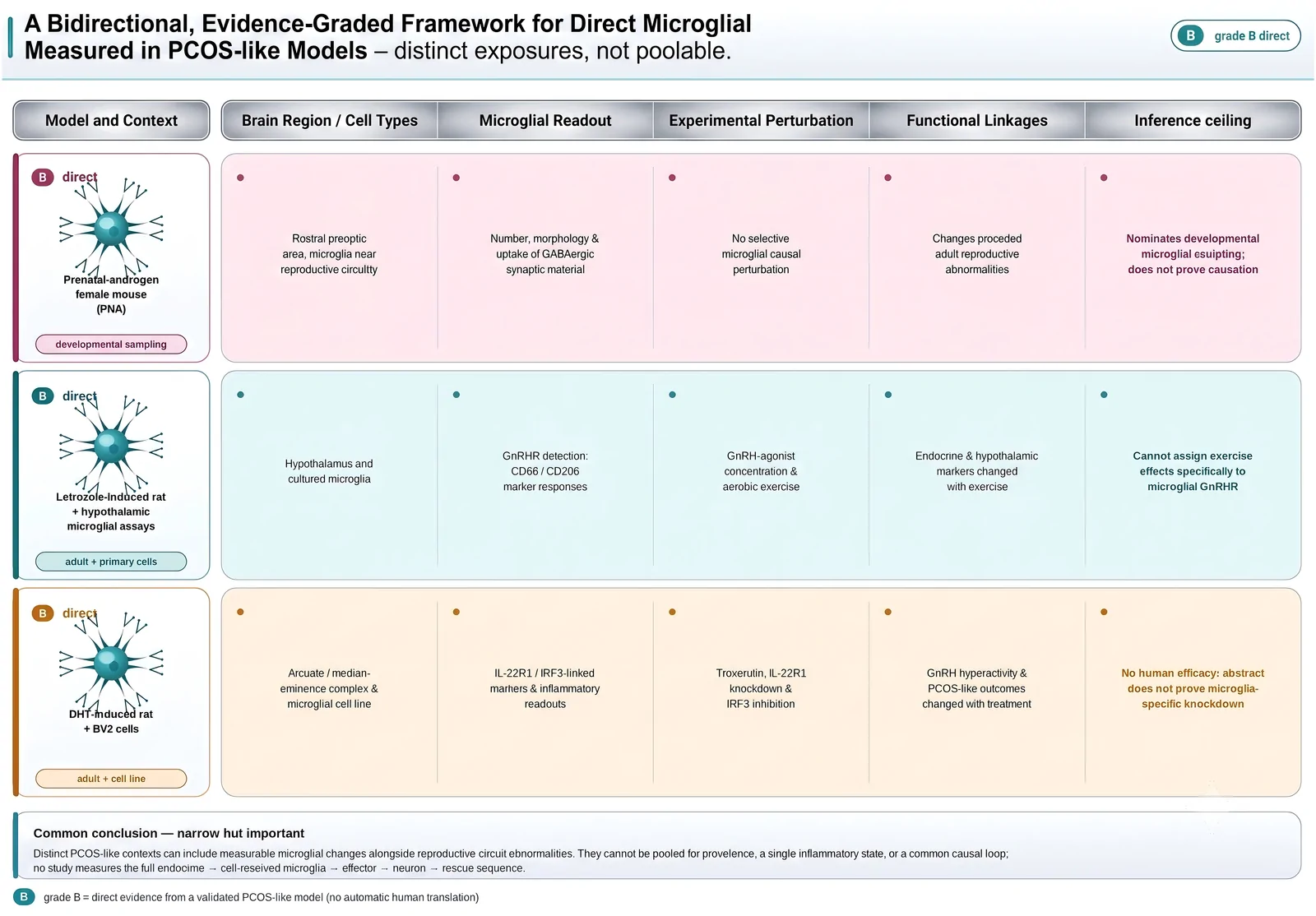
Direct microglial evidence across three non-equivalent PCOS-like models. Each row represents one non-equivalent PCOS-like study. Prenatal androgen exposure was associated with developmental, region-specific microglial changes and reduced uptake of GABAergic material (13). The letrozole model reported candidate microglial GnRHR expression and concentration-dependent CD86/CD206 responses, but did not establish receptor necessity or microglial mediation of exercise effects (14). The DHT model linked troxerutin, IL-22R1/IRF3-associated readouts, microglial markers, and GnRH-related outcomes; publicly accessible information does not establish microglia-specific *in vivo* knockdown (15). Grade B denotes direct evidence in a PCOS-like model and does not permit automatic human translation.

### 3.4 Competing feedbacks and phenotype-dependent timing

The combined evidence argues against a single irreversible feed-forward lesion. Developmental androgen exposure can alter wiring before adult reproductive abnormalities, whereas postpubertal DHT can produce acyclicity and weight gain without faster LH pulses or denser GABA appositions (23, 50). Adult ovarian androgens can maintain GnRH-neuron firing after prenatal programming, but firing, transcript trajectories, potassium currents, transmitter responsiveness, and circulating LH do not always covary (51–54). Chronic GnRH-neuron activation is sufficient to generate selected PCOS-like traits in healthy mice (55), demonstrating a causal neuronal route without proving that microglia initiate it.

Developmental and adult manipulations also answer different causal questions. Adult androgen-receptor blockade may reverse maintenance of an established circuit without identifying the prenatal initiating event (25, 51). Postpubertal DHT tests sustained adult exposure, whereas prenatal androgen and AMH paradigms test programming during distinct developmental windows (44, 50). A valid synthesis should therefore preserve exposure timing and distinguish induction, maintenance, compensation, and treatment response.

Cell-targeted androgen studies further separate reproductive and metabolic branches. Combined brain/adipose androgen-receptor loss prevented many DHT-induced traits, whereas androgen-receptor deletion in leptin-receptor-expressing cells improved reproductive but not metabolic outcomes (56, 57). Phenotype, developmental window, region, and assay should therefore be treated as biological modifiers, and any claim that microglia are required must be tested against direct neuronal and peripheral alternatives.

## 4 Hormone- and metabolism-sensitive microglial states

### 4.1 Steroid and GnRH sensitivity

Microglia can respond to reproductive hormones, but most evidence is grade D and non-PCOS. Mouse microglia express steroid receptors and steroid-converting enzymes; estradiol, progesterone, neurosteroids, glucocorticoids, and androgens alter inflammatory, phagocytic, migratory, or repair-related programs according to receptor, sex, age, dose, region, and challenge (58–73). Opposite androgen effects in endotoxin, demyelination, hemorrhage, and metabolic models illustrate why no hormone can be assigned a universal pro- or anti-inflammatory direction (70–74). In PCOS-like models, GnRHR and IL-22R1/IRF3 are candidate nodes (14, 15), but the reported CD86/CD206 pairs are surface-marker snapshots retained only as historical assay labels, not stable M1/M2 identities.

Local steroid metabolism further weakens a simple serum-hormone-to-cell-state model. Microglial enzymes can transform precursors into receptor-active metabolites, and ERα, ERβ, GPER, androgen-receptor, glucocorticoid-receptor, and mineralocorticoid-receptor pathways can produce different outputs (58, 59, 63, 93–95). Immortalized HMC3 or BV2 responses remain useful for target nomination but cannot establish hypothalamic metabolism *in vivo*. Receptor expression, ligand availability, pulsatility, and intracellular metabolism should be measured in the same region and state before a circulating hormone is assigned a microglial effect.

### 4.2 Metabolic inputs and state transitions

Obesity and metabolic models show that lipids, leptin, insulin, adiponectin, gut-derived signals, and other adipokines can alter hypothalamic microglial programs and neuronal function (75–92). Saturated fat can recruit microglial signaling that contributes to neuronal stress and impaired leptin responses, whereas CX3CL1 manipulation can restore POMC excitability (76, 80). Leptin- and insulin-receptor perturbations, hyperinsulinemia, hypoglycemia, and adipokines produce sex- and context-dependent neural and metabolic effects (77–90). Engineered microglial inflammatory signaling can even improve glucose tolerance (111), showing that an inflammatory-labeled state may be harmful, adaptive, or mixed. These findings nominate metabolic bridges but do not establish a reproductive-axis mechanism in PCOS.

Metabolic signaling is bidirectional rather than uniformly pathogenic. Central insulin can induce inflammatory-associated output, but CX3CR1-dependent microglial signaling can also constrain counterregulatory responses during hypoglycemia (81, 84). Adiponectin, visfatin, apelin, chemerin, and resistin activate different migratory or inflammatory programs (85–90). Myeloid leptin- or insulin-receptor perturbation affects neural projections and metabolism in sex-dependent ways (77–83). Consequently, insulin resistance or obesity is not simply a confounder to remove; it may define a biologically distinct route that must be factorially separated from androgen exposure. **Figure 4** summarizes the hormone- and metabolism-to-microglia mechanistic bridges and their evidence boundaries.

**Figure 4:**
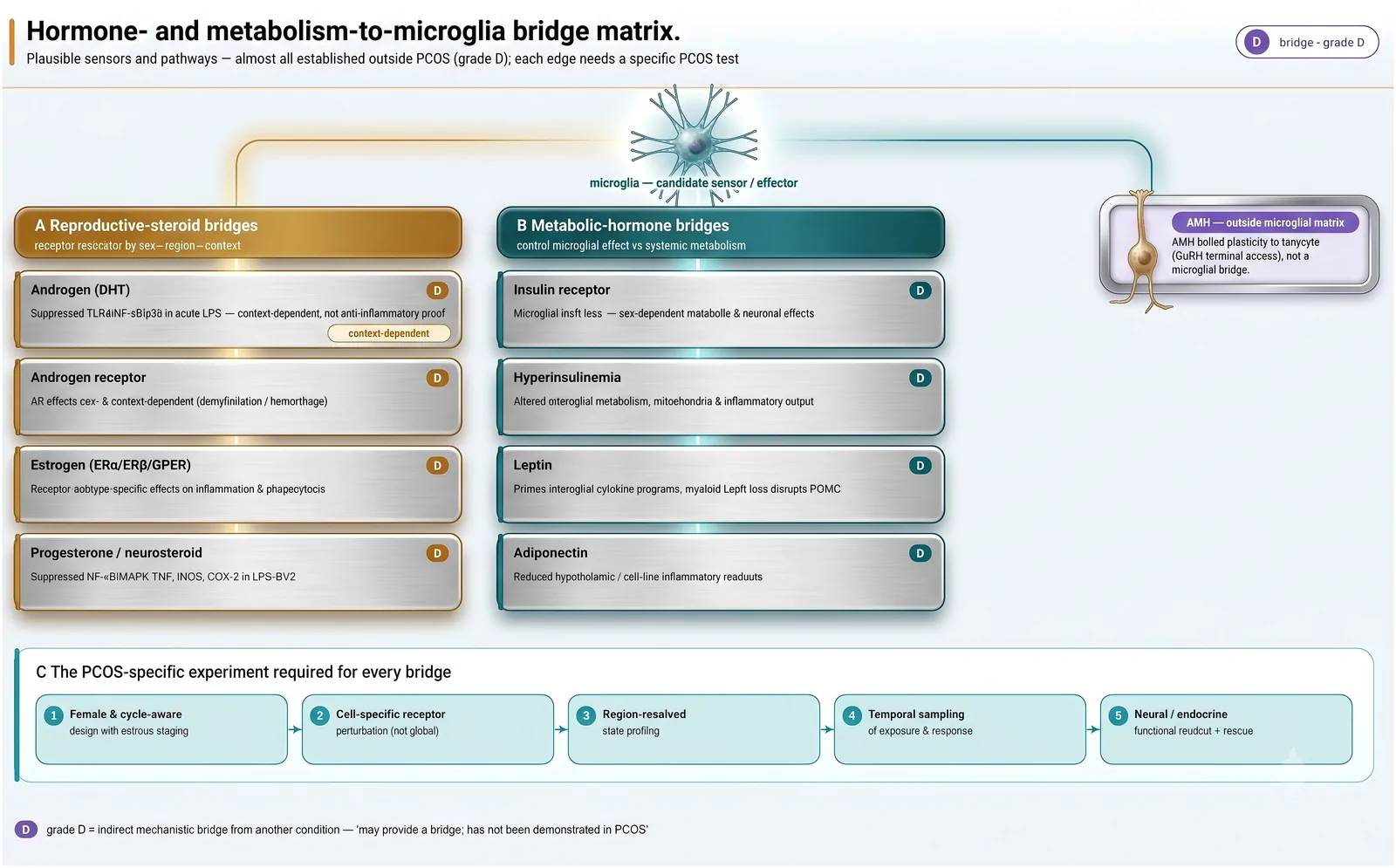
Hormone- and metabolism-to-microglia bridge matrix. Reproductive-steroid and metabolic-hormone studies are arranged as mechanistic bridges rather than direct PCOS evidence. Androgen, estrogen, progesterone, glucocorticoid, insulin, hyperinsulinemia, leptin, and adiponectin effects depend on sex, region, dose, timing, receptor context, and assay (58–71, 76, 80, 82, 83, 86). AMH-linked plasticity is kept outside the microglial matrix because the demonstrated neuroglial interface is tanycytic (12, 44). Every bridge requires a female and cycle-aware PCOS-specific experiment, cell-selective perturbation, regional resolution, temporal sampling, and neural or endocrine rescue. Grade D denotes a non-PCOS bridge.

### 4.3 Cell identity, regional context and state safeguards

Local steroid metabolism and microglial state cannot be inferred from circulating hormone concentration or one marker. Steroid-converting enzymes, ERα/ERβ/GPER pathways, developmental neurosteroid loss, and glucocorticoid exposure produce sex-, region-, and stage-specific effects (59, 63, 93–99). Healthy microglia perform surveillance, transient contacts, complement-linked engulfment, synaptic support, and partial trogocytosis (100–105). The prenatal-androgen finding of reduced GABAergic-material uptake therefore requires functional interpretation rather than an automatic activation label (13). Developmental depletion and perineuronal-net studies provide relevant precedents but also expose CSF1R and systemic confounding (106–108).

A defensible PCOS microglial state requires convergent resident-cell identity, spatial localization, molecular modules, and function. Obesity, primate, cross-species, and neurodegenerative datasets show substantial temporal, regional, cellular, and species variation (109–120). TREM2/APOE programs describe adaptation to lipid and phagocytic stress, not a PCOS-specific label (112–115). Studies should combine lineage-aware markers with spatial methods, morphology, transcript/protein modules, and functional assays; IBA1, CD68, TMEM119, P2RY12, morphology, TSPO, or CD86/CD206 alone cannot establish identity, activation, or mechanism (121–123). At the median eminence, tanycytes, vascular cells, border-associated or infiltrating myeloid populations, nerve terminals, and resident microglia require explicit separation.

State nomenclature should report the measured program rather than assign a binary label. TREM2/APOE-associated lipid handling, proliferative obesity-associated states, surveillance programs, phagocytic stress responses, and cytokine-associated modules may coexist or change over time (109–117). Human and mouse microglia can use different rate-limiting metabolic programs after the same challenge (119, 120). Cross-species names are therefore hypotheses about similarity, not proof of conserved function. Donor-level replication, spatial context, and an orthogonal functional assay are required to interpret a transcriptomic cluster.

## 5 Microglia-neuron communication bridges

### 5.1 Structural remodeling and synaptic surveillance

Microglia can influence circuits through contact, trophic signaling, trogocytosis, and engulfment, but structure does not specify direction. Activity-dependent contacts and developmental complement or phosphatidylserine pathways support context-specific elimination or synaptic support (101–105, 124–128). The direct PCOS-like observation is narrower: prenatal androgen exposure reduced microglial uptake of GABAergic material before adult abnormalities (13). Without selective perturbation, cargo tracing, and rescue, it remains unclear whether this change is pathogenic, compensatory, or developmentally delayed (129, 130).

Complement evidence is strongest in developmental and disease systems outside PCOS. C3/CR3-dependent engulfment and phosphatidylserine recognition can contribute to elimination, but complement components may also participate in tagging, debris clearance, or inflammatory amplification (102, 103, 124–127). A PCOS experiment would need to localize the pathway near GnRH or KNDy elements, demonstrate altered synapse or contact dynamics, perturb the relevant component in microglia, and rescue a neuronal and endocrine endpoint. Measuring C1q or C3 in whole hypothalamus would not complete that chain.

### 5.2 Chemokine, purinergic and cytokine junctions

CX3CL1/CX3CR1 signaling shapes microglial development, surveillance, and neuronal outcomes, but constitutive and acute perturbations answer different questions (131–133). In ovariectomized rats with acute LPS exposure, central CX3CR1 antagonism partly restored LH-pulse measures, providing a reproductive precedent rather than a chronic PCOS model (134). Purinergic routes must also remain separate: P2RY12 supports migration and somatic-junction signaling, CD39/CD73 convert ATP toward adenosine feedback, and P2X7 can promote inflammatory effects in other contexts (135–141). Cytokine and extracellular-vesicle studies add plausible effectors, but astrocytic, neuronal, microglial, and systemic sources must be distinguished (142–145, 166, 167).

Cytokine routes are similarly source dependent. Astrocyte-derived IL-33 can promote microglial engulfment in developmental circuits, whereas TNF, IL-1, IL-6, IL-22, and inflammasome pathways can alter neuronal excitability or tissue inflammation in different models (111, 134, 142–145). Extracellular vesicles can transfer bioactive cargo and modify synaptic function in neurodegenerative systems (166, 167). None of these observations identifies the producing cell, direction, or relevant cargo in PCOS; each pathway requires source-resolved and receptor-specific testing.

### 5.3 Reproductive and metabolic neuronal interfaces

Anatomical and causal precedents outside PCOS show that immune signals can intersect reproductive circuits. Microglial interactions with GnRH neurons and microglial RANK signaling provide non-PCOS precedents for immune–reproductive circuit regulation (146–148). Metabolic studies connect lipid-sensitive or inflammatory microglial programs to POMC/AgRP stress, excitability, leptin signaling, and glucose sensing (76, 79, 80, 87, 91, 111, 143, 149). These studies identify plausible target neurons and assay designs, not evidence that the same routes operate in PCOS.

The RANK-GnRH precedent is important because it demonstrates that an identity-resolved microglial pathway can influence reproductive function, but its disease context is not PCOS (148). In parallel, obesity studies show that microglia can impair or support POMC-neuron integrity and glucose-sensing circuits (76, 80, 111, 143). These examples define the experimental standard for a PCOS edge: direct contact or effector measurement, selective perturbation, simultaneous neuronal physiology, endocrine output, and rescue. Similar anatomy alone is not sufficient.

Accordingly, the three PCOS-model anchors define separate candidate edges: developmental microglial sculpting, a candidate GnRHR sensor, and an IL-22R1/IRF3-associated intervention response (13–15). None completes an endocrine exposure → microglial sensor → effector → reproductive neuron → endocrine output → rescue chain. Candidate microglia–neuron communication mechanisms are summarized in **Figure 5**.

**Figure 5:**
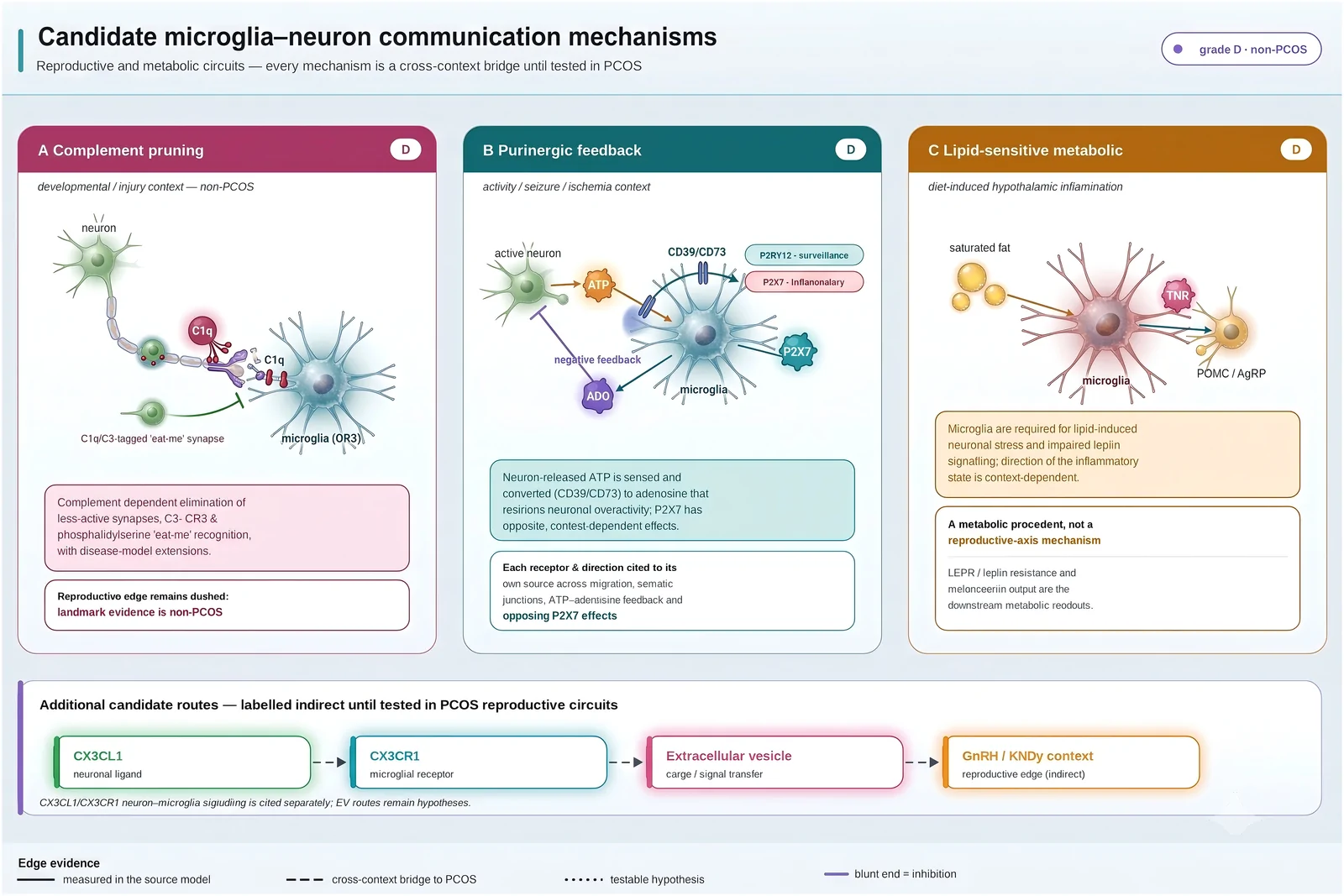
Candidate microglia-neuron communication mechanisms. Panel **(A)** summarizes complement- and phosphatidylserine-associated synaptic elimination in developmental, injury, and disease contexts (102, 103, 124–127). The reproductive-circuit edge remains indirect because these studies are not PCOS studies. Panel **(B)** groups purinergic mechanisms for visual economy but they must not be interpreted as one unidirectional pathway. P2RY12-dependent migration or somatic-junction signaling, CD39/CD73-mediated ATP-to-adenosine feedback, and P2X7-associated inflammatory effects have different directions and source contexts (135–141). Panel **(C)** shows a lipid-sensitive hypothalamic precedent involving microglia, neuronal stress, leptin resistance, and melanocortin output (76). CX3CL1/CX3CR1 and extracellular-vesicle routes remain separately labeled indirect or hypothetical (131, 132, 166, 167). Grade D denotes non-PCOS mechanistic evidence.

## 6 PNETN: triangular interactions and promotion rules

PNETN is most useful as a set of separately testable edges rather than a completed pathway. Evidence is highly asymmetric: human PCOS studies report endocrine and central phenotypes; direct microglial observations come from three heterogeneous PCOS-like studies; and most molecular microglia-neuron mechanisms arise from obesity, inflammation, development, or neurodegeneration. A mechanistic edge must identify the exposure, microglial sensor, intracellular pathway, effector, target neuron, region, timing, and physiological output. Missing elements remain explicit rather than being filled by analogy.

### 6.1 Direct PCOS-model anchors

Three non-equivalent PCOS-like studies provide the direct microglial anchors. Prenatal androgen exposure produced age- and region-specific microglial changes and reduced uptake of GABAergic material before adult reproductive abnormalities (13). The study did not identify a microglial androgen sensor or test microglial necessity. A letrozole-rat study detected a candidate microglial GnRH receptor and concentration-dependent CD86/CD206 responses in cultured hypothalamic microglia (14). Exercise changed endocrine and hypothalamic markers, but receptor dependence and microglial mediation were not established. In a DHT-rat model, troxerutin-associated IL-22/IL-22R1/IRF3 signaling accompanied changes in microglial markers, hypothalamic inflammation, GnRH-related output, and PCOS-like traits (15). Publicly accessible information does not establish that the *in vivo* knockdown was microglia-specific. These records show that model-specific microglial changes can accompany reproductive-circuit abnormalities. They do not define a shared PCOS microglial state or a conserved causal pathway. The three direct PCOS-like microglial studies are compared in **Table 3**.

**Table 3:** Direct PCOS-like microglia studies.

Ref.	Model/timing	Region/cell	Microglial readout	Perturbation	Functional link	Inference ceiling
(13)	Prenatal-androgen female mice; developmental sampling	Rostral preoptic area; microglia near reproductive circuitry	Number, morphology, and GABAergic-material uptake	No selective microglial perturbation	Changes preceded adult reproductive abnormalities	Developmental association only; causation untested
(14)	Letrozole rats; hypothalamic microglial assays	Hypothalamus; cultured microglia	Candidate GnRHR; CD86/CD206 surface-marker responses	GnRH-agonist exposure; aerobic exercise	Endocrine and hypothalamic markers changed	No receptor loss-of-function; exercise mediation unproven
(15)	DHT rats; BV2 assays	Arcuate/median-eminence region; BV2 cell line	IL-22R1/IRF3-associated and inflammatory readouts	Troxerutin; IL-22R1 knockdown; IRF3 inhibition	GnRH-related and PCOS-like outcomes changed	No human efficacy; *in vivo* microglial specificity unconfirmed

The first column lists in-text reference numbers, not PMIDs.

### 6.2 Cross-context mechanistic bridges

Most hormone-to-microglia and microglia-to-neuron mechanisms were established outside PCOS. Microglia can express steroid receptors, metabolize steroid precursors, and alter inflammatory or phagocytic programs after estrogen, progesterone, androgen, insulin, leptin, adipokine, or lipid exposure (58–90). The direction depends on sex, age, brain region, exposure pattern, and experimental challenge. Non-PCOS studies also define candidate effector routes. Microglial RANK signaling can influence GnRH-terminal remodeling and reproductive output (148). Complement-CR3 signaling, phosphatidylserine recognition, CX3CL1/CX3CR1 surveillance, purinergic sensing, ATP-to-adenosine conversion, and cytokine or extracellular-vesicle signaling can modify neuronal structure or activity (103, 124–141, 166, 167). Obesity studies connect lipid-sensitive microglia to POMC-neuron stress, excitability, and leptin signaling (76, 80, 111, 143). These are experimentally informative bridges, not proof that the same edges operate in PCOS reproductive circuits. The principal cross-context mechanistic bridges and the PCOS-specific tests required to validate them are summarized in **Table 4**.

**Table 4:** Mechanistic bridge evidence and required PCOS tests.

Candidate edge	Source context	Supported observation	Evidence grade/directness	Required PCOS-specific test
Microglia-complement-synapse	Postnatal development and mechanistic extensions (102, 103, 124–127)	Microglia engulf synaptic material. C3/CR3 and phosphatidylserine-dependent pathways support context-specific elimination.	D/Indirect	Test complement or CR3 perturbation near GnRH/KNDy neurons in female PCOS-like models.
CX3CL1/CX3CR1-surveillance	Developmental and injury models (131, 132)	Fractalkine signaling shapes microglial development and neuronal outcomes	D/Indirect	Inducible adult perturbation with developmental controls and reproductive endpoints
ATP-adenosine feedback	Activity, somatic-junction, seizure and ischemia models (135–141)	P2RY12-dependent migration or junction signaling, CD39/CD73-mediated ATP-to-adenosine feedback, and P2X7-associated inflammatory effects are distinct, context-dependent mechanisms.	D/Indirect	Test each receptor and direction separately with cell-selective perturbation, reproductive-neuron recording and LH sampling.
Saturated fat-microglia-neuron	Diet-induced hypothalamic inflammation (76)	Microglia are required for lipid-induced neuronal stress and leptin-signaling impairment	D/Indirect	Separate obesity and androgen effects in female PCOS-like factorial models
DHT-microglial inflammatory signaling	LPS neuroinflammation models (70)	DHT suppressed TLR4/NF-kappaB/p38 signaling in that context	D/Indirect	Test direction across dose, sex, region and PCOS-like endocrine context
Microglial insulin receptor	Obese conditional-knockout mice (83)	Microglial insulin-receptor loss produced sex-dependent metabolic and neuronal effects	D/Indirect	Female, cycle-aware conditional perturbation with reproductive outputs
Hyperinsulinemia-mitochondria	HFD mice and microglial cultures (82)	Insulin excess altered microglial metabolism, mitochondrial dynamics and inflammatory output	D/Indirect	Clamp insulin independently of obesity and androgen exposure in PCOS-like models
Adiponectin-microglia	Short-term high-fat feeding and cell assays (86)	Adiponectin reduced hypothalamic and cell-line inflammatory readouts	D/Indirect	Establish central target engagement and microglia-specific mediation in PCOS
AMH-neuroglial interface	Women with PCOS plus mouse tanycyte experiments (12)	AMH associated with human hypothalamic measures and remodeled mouse tanycyte-GnRH access	A/B, Direct but non-microglial	Compare tanycyte- and microglia-specific AMH-receptor perturbations
Androgen receptor-microglia	Demyelination models (71)	Microglial AR expression and androgen effects were sex- and context-dependent	D/Indirect	Inducible microglial AR perturbation in female PCOS-like models with rescue

### 6.3 Alternative directions and promotion criteria

Human PCOS studies constrain the endocrine, neurochemical, imaging, and autonomic outputs that the network should explain, but they do not identify a microglial mediator (6–12, 20, 21). The framework therefore permits several alternatives: hormones may act directly on neurons; hormones or metabolic signals may alter microglia that then modulate neurons; neuronal activity may recruit microglial surveillance; or parallel peripheral signals may affect both cell types. Feedback may be reinforcing, inhibitory, compensatory, or developmentally persistent. An edge should be promoted from association to mechanism only after cell identity, temporal precedence, selective perturbation, a defined effector, an orthogonal neuronal or endocrine endpoint, rescue, and independent replication converge. No current PCOS-like study completes this sequence. The proposed triangle remains an evidence-graded conceptual framework with visible uncertainty, not a demonstrated feed-forward loop.

## 7 Regional and cellular specificity

The meaning of a microglial observation depends on location and developmental stage. The median eminence, arcuate nucleus, rostral preoptic area, hippocampus, and corticolimbic networks differ in barrier properties, neuronal targets, and glial composition; evidence from one compartment cannot be generalized to another without spatial validation.

### 7.1 Hypothalamic and reproductive-circuit interfaces

Bulk hypothalamic inflammatory, GABA, leptin, NLRP3, apoptosis, and methylation findings do not identify a microglial source (45–49). The DHT/IL-22 and letrozole/GnRHR studies provide microglial or primary-cell observations in hypothalamic regions but do not establish a shared state (14, 15). AMH-related median-eminence plasticity involves tanycytes and GnRH-terminal access (12). Arcuate KNDy/GABA and afferent studies report altered expression, connectivity, transmission, or timing alongside preserved endpoints in other models (23, 24, 28, 36, 40 ,41). These findings define candidate target circuits without demonstrating that arcuate microglia initiate them.

The arcuate and median-eminence compartments require especially careful attribution. Tanycytes regulate access between GnRH terminals and portal vasculature, endothelium and perivascular cells shape exposure to circulating signals, and resident microglia occupy nearby parenchymal niches (12). At these interfaces, bulk inflammatory markers or apparent IBA1-positive changes can reflect several cellular sources. Spatial colocalization should therefore be paired with resident-cell lineage evidence and a pathway-specific functional readout.

### 7.2 Preoptic, extra-hypothalamic and autonomic contexts

The rostral preoptic area contains the developmental PCOS-like microglial observation linked to GABAergic-material uptake (13). Nearby GnRH-neuron studies show androgen-maintained firing, altered potassium currents and inputs, and sufficiency of chronic GnRH-neuron activation for selected PCOS-like traits (27, 51, 53, 55). These neuronal results provide a competing direct route rather than proof of microglial initiation. Extra-hypothalamic imaging and animal studies identify corticolimbic, white-matter, hippocampal, and autonomic phenotypes (10, 11, 43, 150–153), but a hippocampal or orbitofrontal signal cannot validate an arcuate reproductive mechanism.

Extra-hypothalamic outcomes should be interpreted as parallel phenotype domains rather than automatically downstream consequences of a reproductive hypothalamic mechanism. Resting-state and diffusion studies report regional or network differences, and letrozole models describe hippocampal inflammatory or cognitive findings (10, 150–153). These signals may reflect metabolic, mood, vascular, autonomic, or treatment-related processes. Connecting them to PNETN requires a region-specific cellular intermediate and an endpoint appropriate to that circuit.

### 7.3 Cell identity at neurovascular and neuroglial interfaces

Cell labels must follow the assay. Direct model studies report morphology or uptake, cultured hypothalamic microglia with tissue markers, or IL-22R1/IRF3-associated readouts (13–15); AMH-linked structural plasticity is tanycytic (12). Astrocyte-derived IL-33 and lipid-loaded astrocyte conditioned media provide non-PCOS examples of astrocyte-microglia communication (142, 154). A defensible assignment requires an anatomically defined region, resident-cell identity, spatial localization of the pathway, and a functional or molecular state readout. This is especially important at the median eminence, where tanycytes, endothelium, vascular/perivascular myeloid populations, nerve terminals, and resident microglia occupy adjacent interfaces.

Border-associated macrophages and infiltrating monocytes are a particular concern at vascular and circumventricular interfaces. Canonical microglial markers may be altered by disease, while broad myeloid markers do not exclude non-parenchymal populations. Studies should document marker combinations, morphology, anatomical position, vascular relationship, and, where possible, lineage or fate mapping. This is necessary before a change near the median eminence is described as a resident-microglial mechanism.

## 8 Peripheral-central coupling and human translational evidence

Peripheral metabolic and inflammatory abnormalities do not prove that a signal reaches or reprograms brain microglia. A complete route must separate the circulating exposure, barrier or neuroglial interface, central responding cell, target neuron, and physiological output. Most mechanistic chains derive from obesity and HFD studies rather than PCOS.

### 8.1 Metabolic, inflammatory and barrier routes

Leptin, insulin, adiponectin, nutrients, and adipose-derived signals alter hypothalamic microglia in non-PCOS models (75, 76, 82, 83, 86). Letrozole and HFD-associated ovulatory-dysfunction models add hypothalamic leptin, inflammatory, and microglial-associated findings, but cell identity, systemic drug effects, and temporal direction remain unresolved (45, 92). Evidence from non-PCOS metabolic models suggests several possible routes linking peripheral metabolic disturbances to the brain. Gut-derived signals and bariatric interventions indicate that gastrointestinal and metabolic changes can influence central physiology, whereas resistin–TLR4 and inflammasome-related pathways illustrate inflammatory routes linking peripheral metabolic disturbances to the brain. Primate and obesity studies further demonstrate that hypothalamic responses are regionally heterogeneous (90, 118, 145, 155–157). However, none of these studies demonstrates that a circulating PCOS-associated signal acts specifically through brain microglia.

Barrier anatomy determines what would count as a peripheral-to-central mechanism. A circulating association cannot demonstrate blood-brain barrier passage, and a hypothalamic response cannot reveal whether the signal arrived through endothelium, tanycytes, circumventricular access, vagal or other neural afferents, or local sensing. Temporal sampling across blood, barrier cells, resident myeloid cells, target neurons, and endocrine output is needed to distinguish these alternatives. Acute LPS-CX3CR1 experiments show that inflammatory signaling can intersect LH-pulse regulation, but acute endotoxemia should not be treated as a chronic PCOS model (134).

### 8.2 Human central, neuroendocrine and autonomic readouts

Human PCOS studies define central endpoints rather than cellular mechanisms. Reproductive physiology varies with protocol, sleep, insulin sensitivity, and menstrual phenotype (6–8, 17–21). CSF GABA/estradiol, resting-state and challenge fMRI, diffusion measures, AMH-associated hypothalamic signals, and autonomic studies support a brain-associated phenotype (9–12, 42, 43, 150, 151, 158–161). Adjustment for insulin sensitivity or adiposity can attenuate cerebral and autonomic differences (11, 159), making clinical stratification essential. LH, CSF analytes, BOLD, MRS/DTI, peripheral autonomic measures, and exercise responses remain indirect with respect to brain microglia.

The human literature contains both positive and null or attenuated findings. Early and later pulse studies differ in amplitude, frequency, metabolic selection, and experimental conditions (6, 7, 17–20). Imaging studies associate cerebral measures with LH, insulin, cognition, emotional symptoms, or autonomic activation, but BOLD and diffusion metrics are not cell-specific (10, 11, 43, 150, 151). CSF gives a central compartment measurement without regional localization (9). Convergence supports brain involvement while leaving the cellular mediator unresolved.

### 8.3 Human brain-microglia gap and clinical heterogeneity

No direct human PCOS brain-microglia study was identified in the cited corpus. Human TSPO imaging cannot fill this gap because TSPO is not microglia-specific and is influenced by genotype, vascular distribution, and cellular heterogeneity (122, 123, 162). Likewise, peripheral cytokines, extracellular vesicles, fMRI, CSF analytes, autonomic measures, and tanycyte remodeling require validated cell-of-origin or target-engagement evidence. The international guideline and human studies emphasize heterogeneity by BMI, insulin resistance, age, medication, reproductive stage, sleep, and psychological phenotype (1). Translation should begin with stratified longitudinal phenotyping and orthogonal cell-proximal assays, not a claim that preclinical microglial pathways already define a treatable PCOS subtype.

A future human study should avoid presenting a single proxy as definitive. A credible design would combine deeply phenotyped and prospectively stratified participants, repeated endocrine and metabolic measurements, a central imaging or fluid signal with known analytical properties, and an orthogonal assay that improves cell-of-origin inference. Even a significant TSPO-PET difference would remain a neuroinflammation-associated signal unless genotype, vascular contribution, ligand kinetics, and comparator conditions were addressed. Human evidence should be used to select endpoints and subgroups, not to back-validate an animal mechanism.

## 9 Model heterogeneity and causal ceiling

PCOS-like models reproduce selected components of a heterogeneous syndrome. Prenatal androgen/AMH, DHT, letrozole, DHEA, metabolic, and comorbidity models differ in exposure window, steroid milieu, species, reproductive output, and brain phenotype; they should be compared, not pooled.

### 9.1 Developmental models

Prenatal androgen models provide developmental circuit evidence for GABA/GnRH/KNDy input, currents, connectivity, and adult androgen dependence, together with important null findings (23, 25, 36, 40, 41, 53). The only direct microglial result in this family is a stage- and region-specific change in rostral preoptic morphology and GABAergic-material uptake (13). Prenatal AMH is distinct: maternal observations are associative, whereas mouse exposure produces persistent GnRH hyperactivity and PCOS-like traits through a neuroglial interface that includes tanycytes (44). Neither exposure represents a universal developmental mechanism.

Positive and null findings within prenatal models should be synthesized together. Denser GABAergic appositions can coexist with unchanged KNDy firing, and adult receptor blockade tests reversibility rather than the original developmental event (25, 36, 40). The microglial phenotype occurs in a restricted region and window, so it cannot be generalized to adult arcuate inflammation (13). Prenatal AMH and androgen exposures should also remain distinct because their maternal context, target interface, and proposed mediators differ (44).

### 9.2 Adult, mixed and comorbidity models

DHT models include reproductive-neuropeptide, bulk epigenetic, androgen-receptor, and IL-22R1/IRF3-associated findings, but intervention response does not identify the initiating lesion (15, 37, 49, 50, 56). Letrozole models show altered LH/KNDy/GnRH responsiveness, kisspeptin-dependent rescue, candidate microglial GnRHR signaling, and bulk hypothalamic inflammatory measures (14, 35, 39, 46–48, 54). DHEA, prenatal-testosterone sheep, LepRb-cell androgen-receptor deletion, seizure, and HFD paradigms emphasize behavioral, neuroanatomical, branch-specific, comorbidity, or metabolic targets (27, 57, 92, 163–165). Model validity must be stated for the endpoint being discussed.

Adult models also separate disease induction from treatment response. A pathway required for benefit from troxerutin or exercise is not necessarily required for DHT or letrozole to induce the phenotype (14, 15). Aromatase inhibition directly changes estrogen availability, while DHT provides non-aromatizable androgen exposure; both can alter neurons, glia, ovary, liver, adipose tissue, and behavior in parallel. Untreated, intervention, and rescue arms should therefore be interpreted as different causal contrasts.

### 9.3 Causal interpretation across models

Model manipulations establish limited causal statements. Chronic GnRH-neuron activation can generate PCOS-like traits and kisspeptin-neuron inhibition can rescue selected letrozole outcomes (39, 55), but these findings establish neuronal rather than microglial causality. The prenatal microglial study is observational (13), the GnRHR study lacks microglial receptor loss-of-function (14), and the IL-22R1/IRF3 intervention has incompletely resolved *in vivo* cell specificity (15). The next step is a factorial, region- and time-restricted microglial perturbation with a defined effector, simultaneous neuronal and endocrine readouts, rescue, and replication in a second non-equivalent model.

Cross-model replication should test generality rather than force convergence. A candidate that alters GnRH activity in letrozole and DHT models may still operate through different upstream exposures or cell states. Conversely, failure to reproduce a pathway may identify a model-specific mechanism rather than invalidate the original result. Prespecified phenotype components and negative controls are essential for deciding which element is transportable to human PCOS.

## 10 Methodological limitations

### 10.1 Search coverage and selection

The search was PubMed-centered and historical executable records, exports, deduplication files, and formal dual-review screening were not retained. The corpus may therefore omit studies indexed differently or located in other databases, and selective citation cannot be excluded. The review should be interpreted as a transparent structured narrative synthesis, not a prevalence estimate, systematic evidence map, or risk-of-bias assessment.

Backward and forward citation checking can reduce, but not eliminate, selection risk in a narrative review. Any update should be reported separately from the historical corpus and should not retroactively create a PRISMA denominator. Reference authenticity, publication status, and claim support are also distinct tasks: a valid DOI or PMID does not establish that the paper supports the cited sentence, and an abstract may omit subgroup, negative, or cell-specific details important to interpretation.

### 10.2 Cell identity, causality and measurement

Human evidence is predominantly cross-sectional or physiologic, and direct microglial evidence is preclinical. IBA1, CD68, morphology, generic gliosis, TSPO, BOLD, MRS, peripheral biomarkers, and bulk hypothalamic measures cannot independently establish a microglial state or causal direction (10, 11, 13–15, 118, 121–123). Cell-line and pharmacologic results require primary-cell and cell-specific validation. Positive intervention responses may reflect peripheral endocrine or metabolic improvement rather than central target engagement.

### 10.3 Model and clinical heterogeneity

PCOS definitions, metabolic phenotype, reproductive stage, sleep, treatment, model trigger, species, brain region, markers, and timing vary across studies (1). Prenatal androgen, DHT, letrozole, AMH, DHEA, and diet models capture different components and cannot be pooled into a universal state. Publication and reporting bias may overrepresent positive pathways, while null findings and model-specific reversals remain essential for interpretation. These limitations restrict completeness and transportability but do not invalidate the central conclusion that direct human microglial evidence is absent and preclinical anchors remain heterogeneous. Key methodological limitations, inferential consequences, and mitigation strategies are summarized in **Table 5**.

**Table 5:** Methodological limitations, inferential consequences and mitigation.

Limitation	Inferential consequence	Minimum mitigation	Residual risk
IBA1 intensity alone	Cannot distinguish cell number, morphology or function	Stereology, morphology, multiple identity/state markers and functional assay	Marker panels remain context-dependent
CD68, TMEM119 or P2RY12 interpreted in isolation	State or identity may be misclassified	Combine lineage/identity markers with transcriptomic and functional evidence	Disease states can alter canonical markers
M1/M2 dichotomy	Collapses heterogeneous and reversible programs	Report measured genes/proteins and unbiased state modules	Cross-study nomenclature remains inconsistent
Uncontrolled estrous stage or hormone exposure	Endocrine-state effects may be mistaken for disease effects	Prespecified cycle staging, dense hormone sampling and factorial design	Staging itself may be perturbed in PCOS-like models
Whole-hypothalamus homogenate	Loses region and cell specificity	Microdissection, spatial methods and cell sorting	Dissociation or sampling biases remain
Different PCOS-like models pooled	Creates a false universal mechanism	Analyze prenatal androgen, DHT, letrozole, DHEA, AMH and diet models separately	No model reproduces all human phenotypes
Peripheral biomarkers used as central proxies	Cannot establish brain entry or microglial action	Paired central/peripheral measures and target-engagement validation	Human tissue access remains limited
BOLD, MRS or TSPO interpreted as microglia-specific	Neurovascular, neuronal, astrocytic or genotype effects may confound	Kinetic modeling, genotype control and orthogonal cell-specific assays	Imaging resolution does not equal cell identity
Cross-sectional human design	Direction and mediation cannot be resolved	Longitudinal sampling, prespecified mediation and negative controls	Residual confounding remains
Non-specific pharmacology	Improvement may arise outside the CNS or outside microglia	Cell-specific perturbation, rescue and systemic target controls	Developmental compensation and off-target effects
Single-cell dissociation and pseudoreplication	Cell proportions and significance can be distorted	Donor-level replication, ambient-RNA correction and mixed models	Rare states may remain underpowered
Publication and selection bias	Positive mechanistic routes may be overrepresented	Preregistration, null-result reporting and protocolized updates	Literature-level bias cannot be eliminated retrospectively

## 11 Technologies and causal validation roadmap

### 11.1 Longitudinal human phenotyping and imaging

The immediate human task is to align dense endocrine sampling, sleep and autonomic monitoring, repeated imaging, and analytically validated fluid measures. Genotype- and kinetic-aware TSPO PET or other neuroimmune tracers may support a central signal but cannot independently establish microglial identity (122, 123, 162). Within-person trajectories, negative controls, and prespecified lagged analyses can strengthen temporal inference, while treatments, irregular cycles, and metabolic confounding require explicit handling (8, 10, 11, 20).

### 11.2 Spatial and cell-identity resolution

Single-cell or single-nucleus sequencing, spatial transcriptomics/proteomics, multiplex imaging, tissue clearing, electron microscopy, and lineage tracing should identify the cell and neighborhood underlying a candidate signal (116–121). The prenatal-androgen uptake phenotype provides a concrete spatial target (13). Inferred clusters or ligand-receptor pairs remain hypotheses until physical interaction, functional readout, and perturbation are demonstrated.

### 11.3 Cell-specific perturbation and human-relevant models

Inducible microglia-restricted deletion, CRISPR perturbation, temporally restricted pharmacology, and rescue can test GnRHR, IL-22R1/IRF3, androgen-receptor, complement, chemokine, and purinergic candidates (14, 15, 138). Simultaneous GnRH/KNDy or metabolic-neuron recording and dense LH sampling should connect target engagement to function. Human microglia-neuron co-cultures, hypothalamic organoids, and barrier systems can model combinations of androgen, AMH, insulin, and inflammatory cues, but maturation, pulsatility, vascular absence, and human-mouse differences remain major design variables (119).

A decisive experiment would require temporally and regionally restricted manipulation of resident microglia in a validated PCOS-like model. Microglial depletion or pathway-specific genetic manipulation should be initiated after establishment of the PCOS-like phenotype to distinguish disease maintenance from developmental programming. GnRH/KNDy neuronal activity, LH pulse dynamics, estrous cyclicity, ovulation, androgen levels, and ovarian morphology should then be measured in parallel.

If microglial manipulation normalizes these outcomes, restoration or reactivation of the candidate microglial pathway should reverse the benefit. Such rescue experiments would provide substantially stronger evidence of causality than changes in IBA1, CD68, cytokines, or hypothalamic bulk expression alone. Replication in a second, non-equivalent model, such as letrozole versus androgen exposure, would determine whether the mechanism is model-specific or generalizable.

### 11.4 Causal integration, biomarkers and reproducibility

CSF and extracellular-vesicle omics may support longitudinal sampling, but cell-of-origin, blood contamination, and analytical reproducibility must be established before a circulating signal is called microglial (166, 167). Prespecified mediation, cross-lagged models, target-trial emulation, and carefully instrumented genetic analyses can compare directional hypotheses but cannot substitute for cellular measurement. A minimum metadata set should capture PCOS phenotype, cycle stage, metabolic status, sex, age, treatment, model validity, region, identity criteria, and raw assay parameters; preregistration, donor-level replication, blinded quantification, shared objects, and independent replication should be planned prospectively (121).

Human-relevant culture systems should be used as reductionist tests rather than replicas of the hypothalamus. Induced-pluripotent-stem-cell-derived microglia, neurons, tanycytes, and endothelial cells can isolate combinations of androgen, AMH, insulin, lipid, and inflammatory signals, but maturity, sex-chromosome background, hormone pulsatility, and missing vascular or peripheral inputs must be documented. A pathway that is reproducible in co-culture should then be tested in a regionally defined *in vivo* model with endocrine output. The proposed technology-to-decision roadmap for causal validation is illustrated in **Figure 6**.

**Figure 6:**
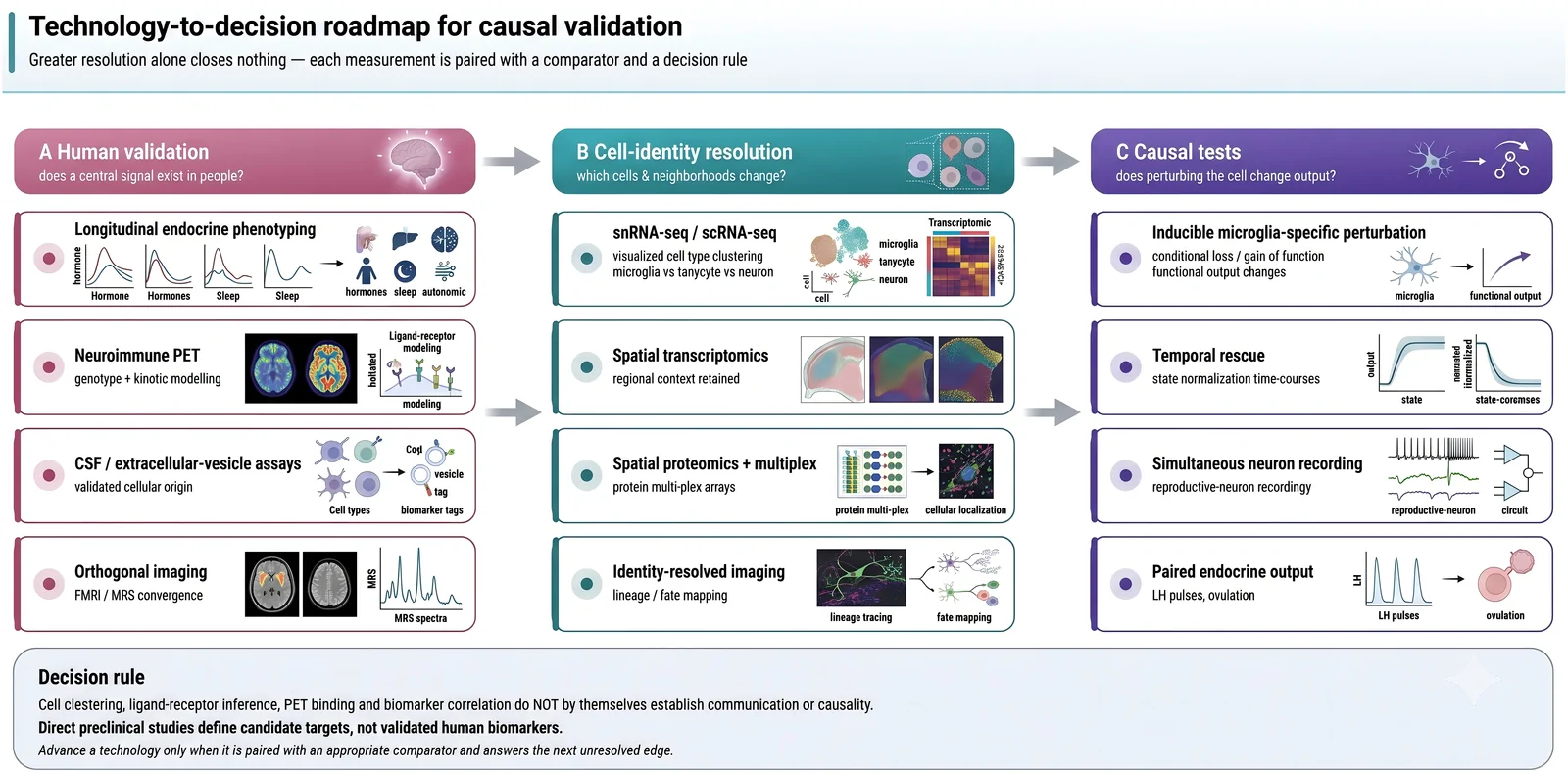
Technology-to-decision roadmap for causal validation. The roadmap is organized into three sequential decision stages: **(A)** Human validation, using longitudinal endocrine phenotyping, kinetic and genotype-aware PET, CSF or extracellular-vesicle assays, and orthogonal imaging to identify central signals, although these approaches do not establish cell identity; **(B)** Cell-identity resolution, using single-cell or single-nucleus sequencing, spatial transcriptomics, spatial proteomics and multiplex imaging, and identity-resolved imaging to localize candidate cells, although these methods do not establish causality; and **(C)** Causal testing, requiring inducible microglia-specific perturbation, temporal rescue, simultaneous neuronal recording, paired endocrine output, and appropriate comparators. Direct preclinical studies nominate targets; they do not validate human biomarkers (13–15).

## 12 Therapeutic implications

Current evidence supplies preclinical targets and human endpoints, not a microglia-directed PCOS treatment base.

### 12.1 What intervention studies show

Troxerutin/IL-22R1/IRF3 and exercise/GnRHR studies nominate pathways in DHT and letrozole models, but neither demonstrates human efficacy or exclusive microglial mediation (14, 15). Butyrate, acetate, electroacupuncture, and other PCOS-like interventions alter bulk hypothalamic, neuronal, endocrine, or inflammatory measures without establishing a microglial source (37, 38, 46–48). Non-PCOS interventions involving CX3CL1, dapagliflozin, gastric bypass, estradiol, testosterone, or engineered inflammatory signaling show that central immune responses can be harmful, compensatory, or mixed (74, 80, 92, 96, 111, 155). They should nominate assays and safety questions rather than therapies.

Directionality is a safety issue. In some obesity paradigms, reducing reactive microglial signaling accompanies metabolic improvement, whereas engineered inflammatory signaling can improve glucose tolerance through neuronal pathways (80, 111). A drug that globally suppresses microglia could therefore remove an adaptive response or alter synaptic maintenance. Therapeutic prioritization requires evidence that the targeted state is maladaptive in the relevant PCOS subgroup, developmental stage, and brain region.

### 12.2 Translation gates and safety

A target should advance only after a reproducible state is localized to a reproductive circuit, shown to precede or modify physiology, perturbed with microglia-specific tools, rescued at cellular and neural/endocrine levels, and reproduced across models. Systemic target engagement must be separated from ovarian, hepatic, adipose, gut, vascular, and neuronal actions. Human imaging and AMH/tanycyte findings provide endpoints but not microglial target validation (10, 12). Other-disease single-cell and cross-species data mainly inform assay design (118, 119). Both intended reproductive effects and potential metabolic harm must be measured.

Developmental stage is a particularly important safety variable for microglia-directed interventions. Microglia participate in synaptic remodeling, circuit maturation, surveillance, trophic support, and tissue homeostasis, and these functions vary across developmental and physiological stages (100–108). Broad depletion or sustained suppression during development or puberty could therefore interfere with physiological synaptic refinement or neuroendocrine circuit maturation rather than selectively correcting a PCOS-associated state (106–108, 121). Even in adults, prolonged suppression may carry risks of disrupting homeostatic surveillance, metabolic adaptation, or responses to injury (100, 101, 121).

Accordingly, future therapeutic studies should distinguish transient state-specific modulation from global microglial depletion. Region-restricted, temporally controlled, and pathway-selective approaches should be prioritized over chronic pan-microglial suppression. Reproductive, metabolic, behavioral, and neurodevelopmental outcomes should be assessed in parallel, particularly when interventions are applied during adolescence or other periods of neuroendocrine remodeling.

## 13 Seven prioritized research questions

The research agenda is condensed to seven non-duplicative questions: (Q1) can a reproducible central neuroimmune-associated signal be identified in stratified human PCOS cohorts; (Q2) which PCOS-like model best reproduces each human central phenotype; (Q3) which candidate microglial hormone-sensing nodes, including AR, GnRHR, and IL-22R1/IRF3, are causal; (Q4) how do tanycytes and microglia contribute separately or jointly to the AMH-GnRH interface; (Q5) which communication mechanism - complement, CX3CL1/CX3CR1, purinergic signaling, or TREM2/APOE-associated metabolism - alters reproductive circuits; (Q6) does temporally controlled microglial perturbation change KNDy/GnRH activity, LH pulses, and ovulation; and (Q7) can a perturbation-validated state support a transportable human biomarker or proof-of-mechanism intervention.

The questions are intentionally staged. Q1 and Q2 establish whether a human signal and an appropriate model exist; Q3-Q6 test cellular sensors, neuroglial interfaces, communication pathways, and causal endocrine output; Q7 is conditional on prior perturbation-validated evidence. This ordering prevents biomarker or treatment development from preceding confirmation of the biological target.

Near-term studies should prioritize human stratification, cross-model comparison, and simultaneous microglial-neuronal-endocrine measurement. Mechanistic promotion requires cell identity, temporal precedence, selective perturbation, rescue, an orthogonal endpoint, and independent replication. **Table 6** specifies models, measurements, causal tests, and evidence boundaries; **Figure 7** shows the qualitative priority structure.

**Table 6:** Seven prioritized research questions.

ID	Question and hypothesis	Recommended model/object	Key measurements	Causal method and main difficulty	Evidence basis
Q1	Can a reproducible central neuroimmune-associated signal be identified in clinically stratified PCOS?	Longitudinal PCOS cohorts stratified by metabolic and reproductive phenotype	Hormones, LH pulses, sleep/autonomic measures, imaging and validated fluid assays	Temporal replication plus orthogonal cell-proximity validation; high difficulty	(9–11, 42, 123, 150, 158–162)
Q2	Which PCOS-like model reproduces each human central phenotype, and which findings are model-specific?	Parallel prenatal-androgen, DHT, letrozole, AMH and metabolic models	Cell states, spatial proximity, engulfment, neural/endocrine and metabolic outputs	Blinded preregistered factorial comparison; high difficulty	(13–15, 23, 35, 44, 50)
Q3	Are candidate hormone-sensing nodes in microglia causal?	Inducible microglial AR, GnRHR and IL-22R1/IRF3 perturbation in female PCOS-like models	Receptor localization, state modules, GnRH/KNDy activity, LH pulses and cyclicity	Cell-specific loss-of-function and rescue; high difficulty	(14, 15, 70, 71)
Q4	How do tanycytes and microglia contribute separately or jointly to the AMH-GnRH interface?	AMH-exposed mice and identity-resolved neuroglial co-cultures	Tanycyte structure, GnRH vascular access, microglial state and LH pulses	Reciprocal cell-specific receptor perturbation and rescue; high difficulty	(12, 44)
Q5	Which candidate communication mechanism alters reproductive circuits?	Female PCOS-like models with spatial profiling and neural recording	Complement, CX3CL1/CX3CR1, P2RY12/P2X7/CD39/CD73, TREM2/APOE, synapses and firing	Pathway-specific perturbation and rescue; high difficulty	(102, 103, 112–115, 124–141)
Q6	Does temporally controlled microglial perturbation alter KNDy/GnRH activity, LH pulses and ovulation?	PCOS-like rodents with simultaneous neural recording and dense endocrine sampling	Microglial state, KNDy/GnRH activity, LH frequency/amplitude, ovulation and metabolic outputs	Region- and time-restricted perturbation with rescue; high difficulty	(8, 13–15, 20, 134, 148)
Q7	Can a perturbation-validated state support a transportable human biomarker or proof-of-mechanism intervention?	Cross-model atlas, independent validation cohort and staged human study	State modules, spatial proteins, fluid markers, imaging, target engagement and symptoms	Hold-out validation, target engagement, and safety analysis; very high difficulty	(118–123, 162, 166, 167)

The seven priorities consolidate overlapping human-cohort, receptor, communication-pathway, causal-output and translation questions. Evidence-basis citations indicate the source of the hypothesis; they do not imply that the mechanism has already been demonstrated in PCOS.

**Figure 7:**
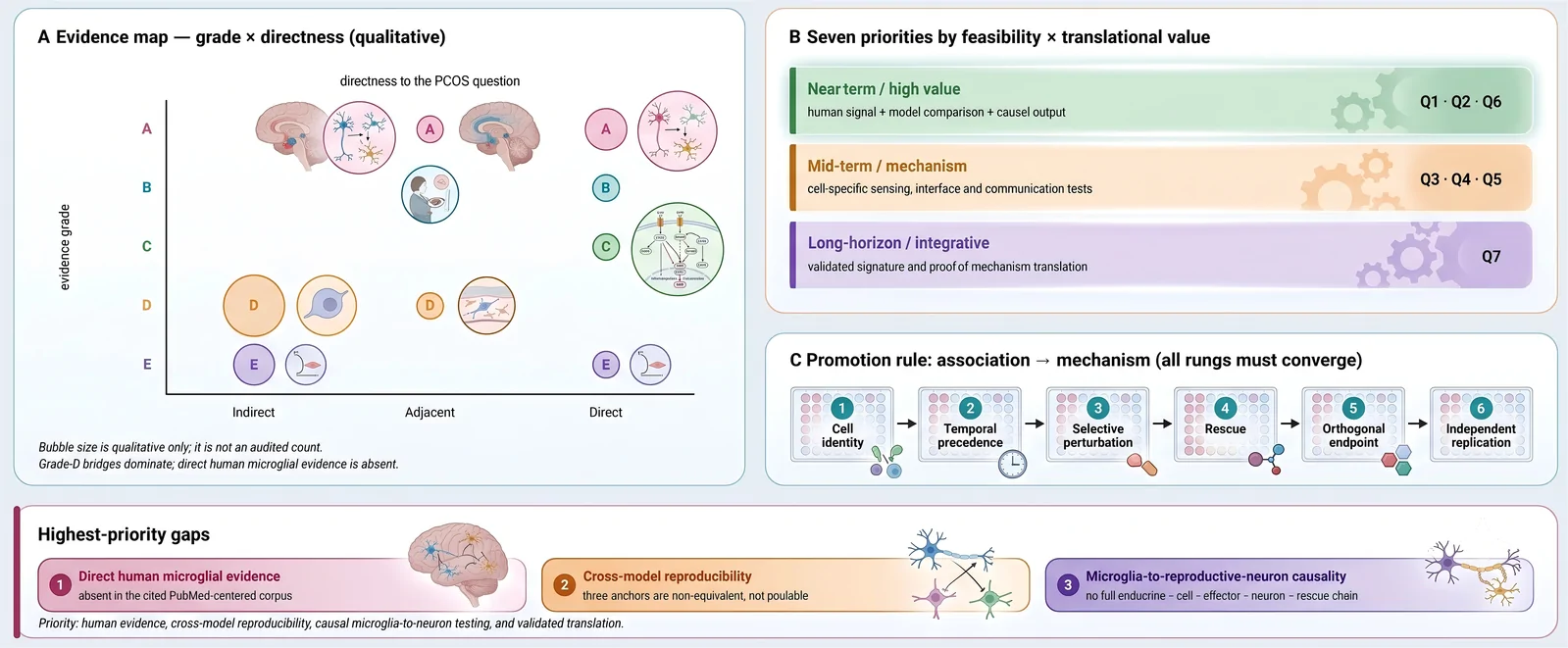
Evidence gaps, experiment prioritization and promotion rules. Panel **(A)** is qualitative; bubble size is not an audited count. Evidence grades describe proximity to the PCOS question rather than study quality, and direct human brain-microglial evidence remains absent in the cited corpus. Panel **(B)** groups seven prioritized questions into near-term human/model work, mid-term mechanism testing, and long-horizon translation. Panel **(C)** requires convergence of cell identity, temporal precedence, selective perturbation, rescue, an orthogonal endpoint, and independent replication before an association is promoted to a mechanism. The highest-priority gaps remain direct human evidence, cross-model reproducibility, and microglia-to-reproductive-neuron causality.

## 14 Discussion and conclusions

### 14.1 Evidence and framework conclusion

The cited corpus contains three direct PCOS-like microglial studies: developmental changes in abundance and GABAergic-material uptake, candidate microglial GnRHR signaling, and an IL-22/IL-22R1/IRF3-associated intervention response (13–15). They establish tractable hypotheses in defined preclinical contexts but do not demonstrate a shared state, conserved causal chain, prevalence in patients, or therapeutic efficacy. No direct study of brain microglia in people with PCOS was identified.

### 14.2 Network interpretation and action

Human PCOS studies report altered endocrine feedback, neurochemistry, cerebral activity, autonomic coupling, and AMH-associated hypothalamic measures, while mechanistic AMH neuroglial remodeling was demonstrated in mouse tanycytes (6–12, 44). Non-PCOS experiments show that microglia can sense hormones and metabolic cues and modify neuronal structure or activity (58, 59, 65, 70, 74, 103, 131, 134, 136, 138, 139, 141, 148, 166, 167). These layers justify a testable PNETN but do not show that every edge operates in PCOS, that one directional loop explains clinical heterogeneity, or that a central immune change is necessarily harmful.

### 14.3 Final conclusion

This structured narrative review improves traceability by separating evidence proximity, directness, cell identity, and causal wording, while acknowledging limitations in search reproducibility and evidence coverage. The decisive next step is not broad anti-inflammatory treatment. It is to identify a human-relevant microglial state, localize it to a reproductive circuit, establish temporal order, perturb it with cell-specific tools, rescue an orthogonal neural or endocrine endpoint, and reproduce the result across models. Until those conditions converge, microglia should be treated as plausible and experimentally accessible contributors to PCOS neurobiology, not established clinical targets.

The central scientific tension should remain visible: direct neuronal mechanisms can be sufficient in selected models, while microglial pathways remain plausible modifiers whose necessity is untested. PNETN is useful only if it accommodates this competition and allows an edge to be rejected. A negative cell-specific perturbation, a model-specific effect, or failure of rescue would refine the network rather than represent an unsuccessful experiment.
