# Supplementary material for: Microglia as candidate links between hormonal signals and neuronal circuits in polycystic ovary syndrome: evidence gaps and a research agenda
Source: Front Endocrinol (Lausanne). 2026 Oct 2;17:1952469. doi: 10.3389/fendo.2026.1952469 (PMC13673313; doi:10.3389/fendo.2026.1952469)
Supplement: Supplementary file 1 [file Table1.docx]

# **Supplementary Table S1. Historical search domains and reproducibility status**

*Note. The table records topic domains and limitations only. Search strings, timestamps, exports, and complete screening logs were not retained; therefore, hit counts and candidate denominators are not reported.*

| **Domain** | **Purpose** | **Discovery source** | **Executable string status** | **Count status** | **Boundary** |
| --- | --- | --- | --- | --- | --- |
| P1 | Direct PCOS-microglia studies | PubMed | Not retained | Not reported | Corpus scope only |
| P2 | PCOS central neuroimmune findings | PubMed | Not retained | Not reported | Cell identity required |
| P3 | PCOS neuroendocrine circuits | PubMed | Not retained | Not reported | Broad, potentially ranking-sensitive |
| P4 | Human PCOS central phenotypes | PubMed | Not retained | Not reported | Not microglia-specific |
| P5 | PCOS animal and developmental models | PubMed | Not retained | Not reported | Models analyzed separately |
| P6 | Reproductive hormone-microglia mechanisms | PubMed | Not retained | Not reported | Mostly non-PCOS bridges |
| P7 | Metabolic hormone-microglia mechanisms | PubMed | Not retained | Not reported | Mostly obesity/metabolic contexts |
| P8 | Microglia-neuron communication | PubMed | Not retained | Not reported | Cross-context evidence |
| P9 | PCOS central methods and translation | PubMed | Not retained | Not reported | Methodological mapping |
| P10 | PCOS definitions, guidelines and reviews | PubMed | Not retained | Not reported | Framing only |
| S1-S3 | Targeted bridge and landmark curation | PubMed | Not retained | Not reported | Not independent systematic searches |
| Crossref | Selected DOI metadata comparison | Not a discovery source | Not applicable | Not applicable | No screening denominator |
| Other databases | Web of Science, Scopus, Embase | Not searched | Not applicable | Not applicable | Coverage incomplete |
